# Gene–Environment Interactions in Face Categorization: Oxytocin Receptor Genotype x Childcare Experience Shortens Reaction Time

**DOI:** 10.3389/fpsyg.2022.873676

**Published:** 2022-06-09

**Authors:** Michelle Jin Yee Neoh, Peipei Setoh, Andrea Bizzego, Moses Tandiono, Jia Nee Foo, Albert Lee, Marc H. Bornstein, Gianluca Esposito

**Affiliations:** ^1^Psychology Program, School of Social Sciences, Nanyang Technological University, Singapore, Singapore; ^2^Department of Psychology and Cognitive Science, University of Trento, Rovereto, Italy; ^3^Lee Kong Chian School of Medicine, Nanyang Technological University, Singapore, Singapore; ^4^Human Genetics, Genome Institute of Singapore, Singapore, Singapore; ^5^Eunice Kennedy Shriver National Institute of Child Health and Human Development, Bethesda, MD, United States; ^6^Institute for Fiscal Studies, London, United Kingdom; ^7^UNICEF, New York, NY, United States

**Keywords:** face categorization, perceptual expertise, multiracial, oxytocin receptor gene, gene–environment interaction

## Abstract

Human faces capture attention, provide information about group belonging, and elicit automatic prepared responses. Early experiences with other-race faces play a critical role in acquiring face expertise, but the exact mechanism through which early experience exerts its influence is still to be elucidated. Genetic factors and a multi-ethnic context are likely involved, but their specific influences have not been explored. This study investigated how oxytocin receptor gene (OXTR) genotypes and childcare experience interacted to regulate face categorization in adults. Information about single nucleotide polymorphisms of OXTR (rs53576) and experiences with own- and other-race child caregivers was collected from 89 Singaporean adults, who completed a visual categorization task with own- versus other-race faces. Participants were grouped into A/A homozygotes and G carriers and assigned a score to account for their type of child caregiver experience. A multivariate linear regression model was used to estimate the effect of genetic group, child caregiver experience, and their interaction on categorization reaction time. A significant interaction of genetic group and child caregiver experience (*t* = 2.48, *p* = 0.015), as well as main effects of both genetic group (*t* = −2.17, *p* = 0.033) and child caregiver experience (*t* = −4.29, *p* < 0.001) emerged. Post-hoc analysis revealed that the correlation between categorization reaction time and child caregiver experience was significantly different between the two genetic groups. A significant gene x environment interaction on face categorization appears to represent an indirect pathway through which genes and experiences interact to shape mature social sensitivity to faces in human adults.

## Introduction

Facial characteristics are employed in the perception and categorization of individuals into groups (Macrae and Quadflieg, 2010). People can accurately identify the social categories to which individuals belong, such as age, race, and sex (Zhao and Bentin, 2008), by using specific physiognomic cues (see Rule and Sutherland, 2017). For example, face categorization decisions regarding race are influenced by skin color (see Maddox, 2004 for review), face shape (Hill et al., 1995), and other facial features, such as nose shape, lip fullness, hair texture, and quality and jaw width (e.g., Blair and Judd, 2011; Stepanova and Strube, 2012; Strom et al., 2012; Dunham et al., 2015). Hence, learning indicative perceptual features of group membership constitutes an effective component of categorization accuracy (Rule and Sutherland, 2017). Individuals with more interactional experiences with members of a particular social category categorize faces more accurately, possibly due to their more frequent opportunities to learn relevant facial features that distinguish social groups (Rule et al., 2010; Brambilla et al., 2013).

Real-life experiences have been proposed to play a critical role in acquiring face expertise (Lee et al., 2011) where an individual’s ability to categorize faces based on social concepts, such as race, stems from early repeated experiences and develops through socialization (Rutland et al., 2005; Dunham et al., 2013; Xiao et al., 2015). For example, 3- and 4-month-old infants show better recognition for faces of the same race (and gender) of their primary caregiver, and 8- and 9-month-old infants with female other-race child caregivers show better recognition for other-race faces compared to infants without such experience, suggesting roles of caregiver experience and other-race contact for face processing (Tham et al., 2018). Moreover, 9-month-old White infants categorize and individuate White faces but not Chinese faces, pointing to the role of experience in categorizing face race (Anzures et al., 2010). Thus, current evidence indicates that experience with a group and early caregiver experience with other-race caregivers can influence infants’ distinctions between members of different social categories.

Besides the effects of experience with faces on face processing, genetic factors are associated with social cognition and social sensitivities (see Skuse and Gallagher, 2011). We propose that such genetic factors could influence race-based face categorization. A potential gene of interest in this connection is the oxytocin receptor gene (OXTR). The oxytocin system has been implicated in human socially related personality traits and behaviors (Donaldson and Young, 2008; Meyer-Lindenberg et al., 2011). Specifically, the OXTR has been identified as a candidate gene regulating attachment-related behaviors and social cognition in humans where the link between single nucleotide polymorphisms (SNPs) in this gene and sociality has been examined (Gillath et al., 2008; Ebstein et al., 2010). One such SNP is rs53576 (G/A). Compared to A allele carriers, G allele homozygosity (GG) is associated with higher (i) self-reported empathy (Rodrigues et al., 2009), (ii) prosocial features (Tost et al., 2010), and (iii) general sociality as rated by peers (Kogan et al., 2011). By contrast, A allele carriers appear to have social information processing deficits in, for example, emotion recognition (Rodrigues et al., 2009). Previous studies have also examined gene–environment interactions between OXTR genotypes and environmental factors on responses to social stimuli (Cataldo et al., 2020), sociability (Carollo et al., 2021), and psychopathology (Cataldo et al., 2018; Zhang et al., 2021), suggesting that OXTR genotypes may also interact with environmental factors in influencing face categorization, which involves the processing of social information relating to faces. For example, rs53576 A/A carriers with high levels of paternal care showed less adaptive responses to social stressors (Cataldo et al., 2020) and followed more Instagram profiles (Carollo et al., 2021), indicating differences between OXTR genotypes in their response to social stressors and social media activity. The review by Cataldo et al. (2018) found that both rs53576 and rs2254298 were significantly involved in gene–environment interactions with early parental care in modulating psychopathology risk. In a similar vein, Zhang et al. (2021) found that higher childhood adversity was associated with depressive symptoms only in rs2254298 G/G carriers in a sample of incarcerated males although such a gene–environment interaction was not observed for rs53576.

Oxytocin appears to improve face processing, such as facial emotion recognition (Guastella et al., 2008; Bartz et al., 2011; Lopatina et al., 2018), and administration of oxytocin facilitates face recognition (Bate et al., 2015) and face memory (Rimmele et al., 2009). rs53576 genotypes have previously been found to be associated with implicit responses to infant and adult faces, where G/G carriers exhibited a more positive implicit association to ingroup infant faces (Senese et al., 2017). The rs53576 genotype has also been associated with neural responses toward ingroup and outgroup faces where A/A carriers showed significant empathic neural responses for faces with both shared racial and group identity compared to G/G carriers who showed such empathic responses for faces with either shared racial or group identity (Luo et al., 2019), suggesting that OXTR rs53576 may interact with intergroup relationships on prosocial behavior. However, evidence about associations between SNPs and face processing is inconsistent. For example, one study reported a significant association between face recognition and the OXTR SNP rs237887 (Skuse et al., 2014), whereas another study failed to find significant associations between 17 OXTR SNPs, including rs237887, and face processing (Verhallen et al., 2017). In a third study, face recognition parameters were associated with OXTR SNPs, where rs53576 and rs2254298 were proposed to lead to poorer performance across social cognition measures, including face recognition (Slane et al., 2014).

**Figure 1:**
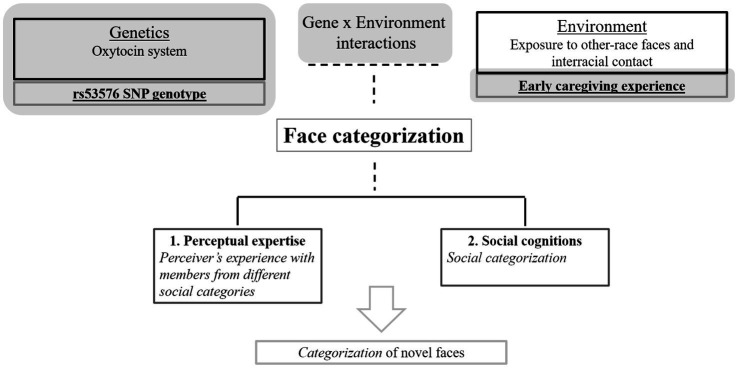
Diagram of key research variables of the present study highlighted in grey in the figure.

The present study aims to explore how genetic factors, childhood experience with other-race child caregivers, and gene × experience interactions influence mechanisms involved in race-based face categorization in a multiracial context. Specifically, we investigated the effects of OXTR rs53576 genotype and early caregiver experiences with either own- or other-race child caregivers in Singaporean adults’ categorizing faces by race (Figure 1). The rs53576 SNP genotype was examined as the G allele appears to be associated with prosocial traits including sociality and social cognition (Tost et al., 2010; Kogan et al., 2011; Li et al., 2015), and has been previously implicated in responses to ingroup/outgroup faces (Senese et al., 2017; Luo et al., 2019) which is related to the present study on own vs. other-race face categorization. Finally, the effect of rs53576 genotype on face categorization has not been studied previously.

The times taken for visual categorizing of face stimuli by race were measured. In line with previous findings (Tham et al., 2018; Woo et al., 2020), we expect individuals with other-race early child caregiver experience to show faster categorization times than individuals with own-race early child caregiver experience or no experience. However, G carriers may be more likely to have greater social interactions and contact with others than A/A carriers, possibly resulting in exposure to child caregivers during early childhood to constitute a more significant source of interracial contact for A/A carriers compared to G carriers. Hence, starting from the idea that early caregiver experience would interact with OXTR genotype in influencing face categorization (operationalized as response times to face stimuli of own and other-race in our study), the following hypotheses were investigated:

*Hypothesis 1*: It is hypothesized an interaction effect between OXTR genotype and early child caregiver experience. Specifically, we expect to observe faster categorization times in individuals with a OXTR genotype A/A and history of other-race child caregivers when responding to own and other-race faces compared to individuals with a history of an own-race child caregiver and G carriers or no experience (both OXTR genotype A/A or G carriers).

*Hypothesis 2*: We expect a main effect of early child caregiver experience. Specifically, individuals with a history of other-race child caregivers will have faster categorization times compared to those with a history of an own-race child caregiver or those with no experience when responding to own and other-race faces.

## Materials and Methods

### Participants

Participants [*N* = 89, Age (years) M = 21.85, SD = 1.70, females = 65] were undergraduates compensated with course credit. All participants were ethnic Chinese. The study was approved by the Nanyang Technological University IRB (IRB Number: IRB-2015-08-020-01) and the research conducted in this study was performed in accordance with Helsinki Declaration. Written informed consent was obtained from participants prior to commencement of the study.

### Procedure

Participants were assessed individually when they were asked to complete a demographics questionnaire at the beginning of the study to obtain information regarding their early experiences with own- or other-race child caregivers. Participants were asked for the following details: (i) age at the time of the child caregiver experience, (ii) duration of the child caregiver experience, and (iii) nationality of their child caregiver. 53 participants experienced non-parental child caregiving. 14 participants had own-race child caregivers, of which 6 were a relative; the rest had other-race child caregivers who were either Indonesian (*n =* 14) or Filipino (*n =* 10). 15 participants had more than one child caregiver who were either Indonesian and Filipino (*n =* 8) or other nationalities (*n =* 7).

After the questionnaire, participants completed a visual categorization task to measure their performance in categorizing faces by race. The task was conducted using a Microsoft Surface Pro tablet with a touch screen, using E-prime 2.0 Professional (Psychology Software Tools, Sharpsburg, PA). Participants were instructed to place their hands at the side of the tablet to standardize the time needed to move their thumb to the response keys. Participants were also reminded to read the instructions carefully and perform the task as fast and as accurately as possible.

### Experimental Task

#### Face Stimuli

To assess participants’ categorizing ability in identifying the race of faces, 4 unique Chinese faces and 4 unique Javanese faces were used. The faces were selected from an existing face database (Yap, Chan, and Christopoulos, 2016). Indonesian Javanese faces were used to represent other-race child caregivers in Singapore as Indonesians made up the majority of foreign domestic workers in Singapore—about 50.4% of foreign domestic workers (Devaraj, 2020). All faces displayed neutral expressions in color and frontal views presented on a white background. The face images were standardized at 480 pixels (17 cm) wide and 600 pixels (21 cm) high with a resolution of 72 pixels per inch and were processed to be the same elliptical shape and size, with eyes and nose centrally aligned.

#### Trials

**Figure 2:**
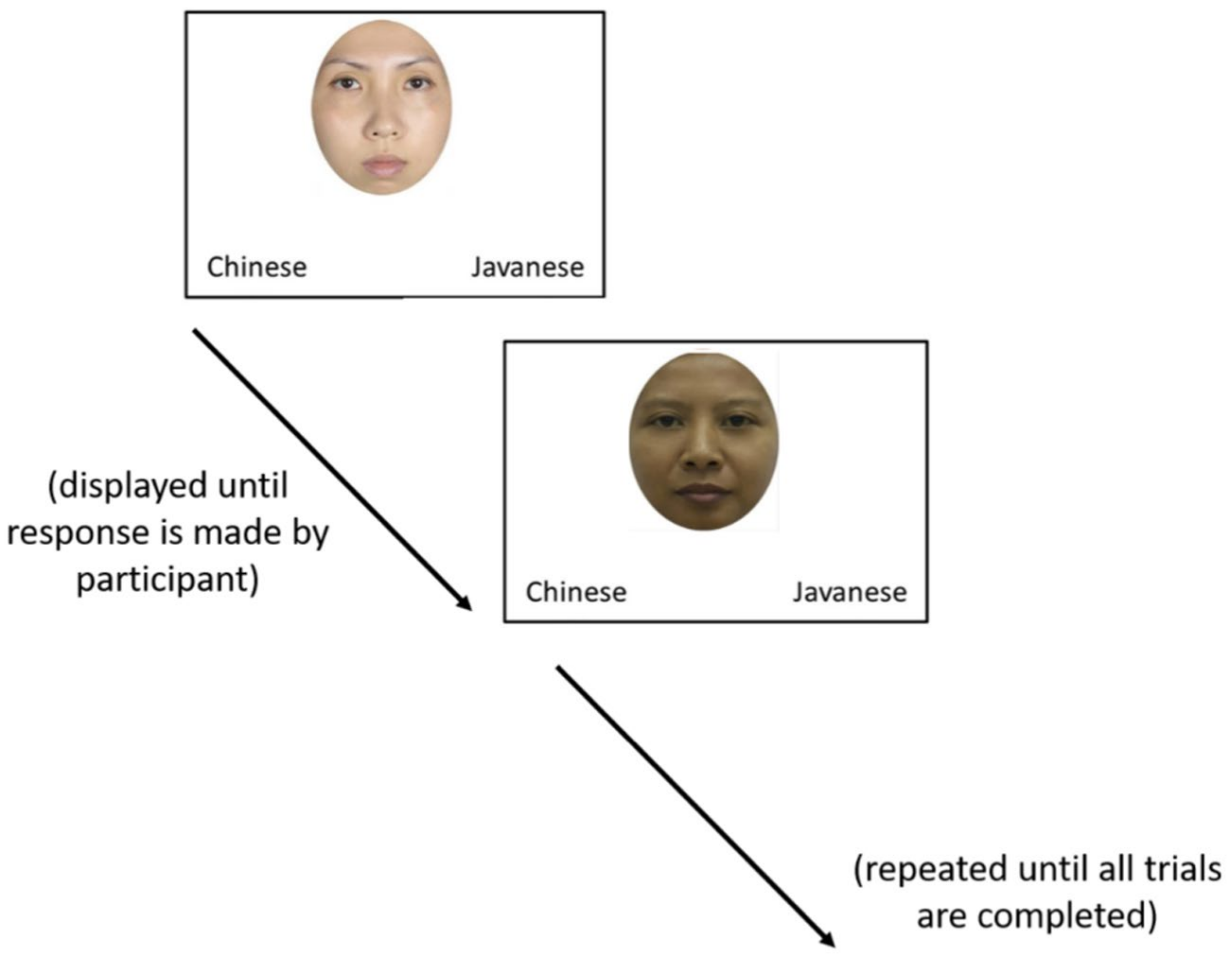
Diagram of experimental setup.

The visual categorization task consisted of eight test trials where either an own- (Chinese) or other (Javanese)-race face stimulus was presented in the middle of the display screen with two race labels, similar to the experimental paradigm employed in Setoh et al. (2019) (Figure 2). These race labels were programmed at the bottom-left or bottom-right corners of the screen as response keys. Participants were instructed to identify the race of the face stimulus by tapping on the response keys. Each face was displayed until the participant made their response by tapping on either response key and a new face was presented following the participant’s response. The face races were presented in a randomized order. To control for and minimize the effect of side preference, the position of race labels was counterbalanced such that the race labels alternated between left and right positions for each race. For instance, half of the own-race face stimuli were programmed with other-race label on the left and own-race label on the right, and vice versa for the other half. The same was programmed for Javanese face stimuli. Participants were not provided with feedback indicating whether their response was correct during the test trials.

Diagram of experimental setup. Figures reproduced from Nanyang facial emotional expression [N-FEE] database (Yap, Chan, and Christopoulos, 2016).

### OXTR Genotyping

Buccal mucosa samples were collected from each participant and genotyped by a laboratory. DNA extraction was performed for each participant using the Oragene DNA purifying reagent and concentrations were assessed using spectroscopy (NanoDrop Technologies, United States). Polymerase Chain Reaction (PCR) was conducted to amplify the target OXTr gene region rs53576 using 1.5 ll of genomic DNA from the test sample, PCR buffer, 1 mM each of the forward and reverse primers, 10 mM deoxyribonucleotides, KapaTaq polymerase, and 50 mM MgCl2. The forward and reverse primers used were 5-GCC CAC CAT GCT CTC CAC ATC-3 and 5-GCT GGA CTC AGG AGG AAT AGG GAC-3. The PCR process involved (i) 15 min of denaturation at 95 degrees Celsius, (ii) 35 cycles at 94 C (30 s), 60 C (60 s), 72 C (60 s), and (iii) 10 min of protraction at 72 C.

Previous studies investigating rs53576 genotypes showed a higher prevalence of G/G homozygotes particularly in European American samples where there tends to be a skewed distribution of A/A, A/G, and G/G genotypes corresponding to 12, 38, and 50%, respectively (Kim et al., 2010). Due to this skewed distribution, previous publications have largely clustered the genotype variants into G/G vs. A/ carriers. However, allele frequencies and linkage disequilibrium patterns differ between Asians and Caucasians (Tost et al., 2011) and multiple studies have found that A allele frequency of rs53576 is higher in Asian populations (Luo and Han, 2014; Butovskaya et al., 2016). The averaged distribution of the different genotypes in the Asiatic population is 45–65% for A/A homozygotes and 35–55% for G carriers (1,000 Genomes project, BioSamples: SAMN07486027-SAMN07486024, dbSNP, 2017).

Participants were therefore classified into “G”: G carriers—those with at least one G allele (G/G homozygotes or A/G) or “AA”: A/A homozygotes. The division of participants into the rs53576 genotypes was: 46 (51.7%) A/A carriers, 30 (33.7%) A/G carriers, and 13 (14.6%) G/G carriers.

### Experience With Child Caregiver

Participants were assigned to three groups, based on the reported experience with child caregivers: (i) No-experience group (*N* = 36): participants who did not receive non-parental caregiving experience; (ii) Own-race group (*N* = 14): participants who received own-race child caregiving; (iii) Other-race group (*N* = 39): participants who received other-race child caregiving.

As experience and interactions with others is posited to provide more opportunity for learning relevant facial features which are distinct between members of different social categories (in this case, race), an individual with an other-race child caregiver can be considered to have more experience with members of that particular social category compared to an individual without an other-race child caregiver. Because all participants in the study were Chinese, individuals with an other-race child caregiver would have more experience with distinguishing relevant facial features of members from a different social category compared to individuals with a same-race child caregiver.

Hence, a new variable (Experience), was created based on the early child caregiving received by participants, in order to account for the different extent of exposure that individuals had with faces of different social categories based on their early caregiver experience: No-experience was assigned an Experience value of 0, Own-race was assigned a value of 1, and Other-race was assigned a value of 2.

### Analytic Plan

Preliminary analysis was conducted to ensure that the independent variable used in this study was appropriate to measure subjects’ face categorization performance and normally distributed. A chi-square test was used to ensure the observed frequencies in the rs53576 genotypes were comparable with those of the reference East-Asian population. Similarly, another chi-square test was used to ensure the observed frequencies in the Experience groups were comparable between the two Genetic Groups (A/A and G carriers). Descriptive and inferential analysis were used to investigate the role played by Genetic Group and Experience on the categorization performance. Specifically, a multivariate linear regression model was fit using an Ordinary Least Square estimator. Then, the main effects for Genetic Group and Experience were investigated using post-hoc analysis: Welch’s t-test was used to assess if there are significant differences between the two genetic groups; Spearman correlation test was used to test if Experience is associated with the categorization performance. Finally, Spearman correlation between Experience and categorization performance was separately computed on the two genetic groups, and a z test was used to test if the two correlations were significantly different. The dataset used in this study can found at Esposito and Setoh (2020).

## Results

### Preliminary Analyses

Prior to data analysis, we first verified that there were no significant differences in the accuracy of categorization responses for Chinese and Javanese faces. The accuracy of the categorization responses was high across participants and no significant differences in the accuracy for Chinese and Javanese faces were found (Wilcoxon test, *W* = 255, *p* = 0.279). We then verified that no differences existed between the RTs of the two races. Since the RT were not normally distributed for both Chinese (M = 1081.0 ms, SD = 260.9 ms, Shapiro–Wilk test: *W* = 0.95, *p* = 0.001) and Javanese (M = 1048.3 ms, SD = 229.1 ms, Shapiro–Wilk test: *W* = 0.90, *p* < 0.001) faces, we tested the difference using a Wilcoxon signed-rank test. No significant difference was found: (*W* = 1,671, z = −1.35, *p* = 0.174, Common Language Effect Size: 0.54). Since no difference between the two races was found, the overall categorization RT was computed for each participant by averaging the RT across all trials. The resulting overall categorization RT was not normally distributed (Shapiro–Wilk test: *W* = 0.95, *p* = 0.001). We then computed the log-transformed overall categorization RT (ORT), which was normally distributed (Shapiro–Wilk test: *W* = 0.98, *p* = 0.126). No significant difference, X^2^(*N* = 89, 2) = 4.52, *p* = 0.104, Cramer’s V = 0.09, was observed between the distribution of participants of this study (A/A: 51.7%, A/G: 33.7%, G/G: 14.6%) and the reference East-Asian population (A/A: 42.1%, A/G: 45.8%, G/G: 12.1%; Yates et al., 2020). The distributions of the participants with different child caregiver experiences were similar between the two genetic groups: *X*^2^(*N* = 89, 2) = 0.527, *p* = 0.768, Cramer’s *V* = 0.08. No differences in age and gender emerged between the groups.

### Descriptive and Inferential Analysis

**Figure 3:**
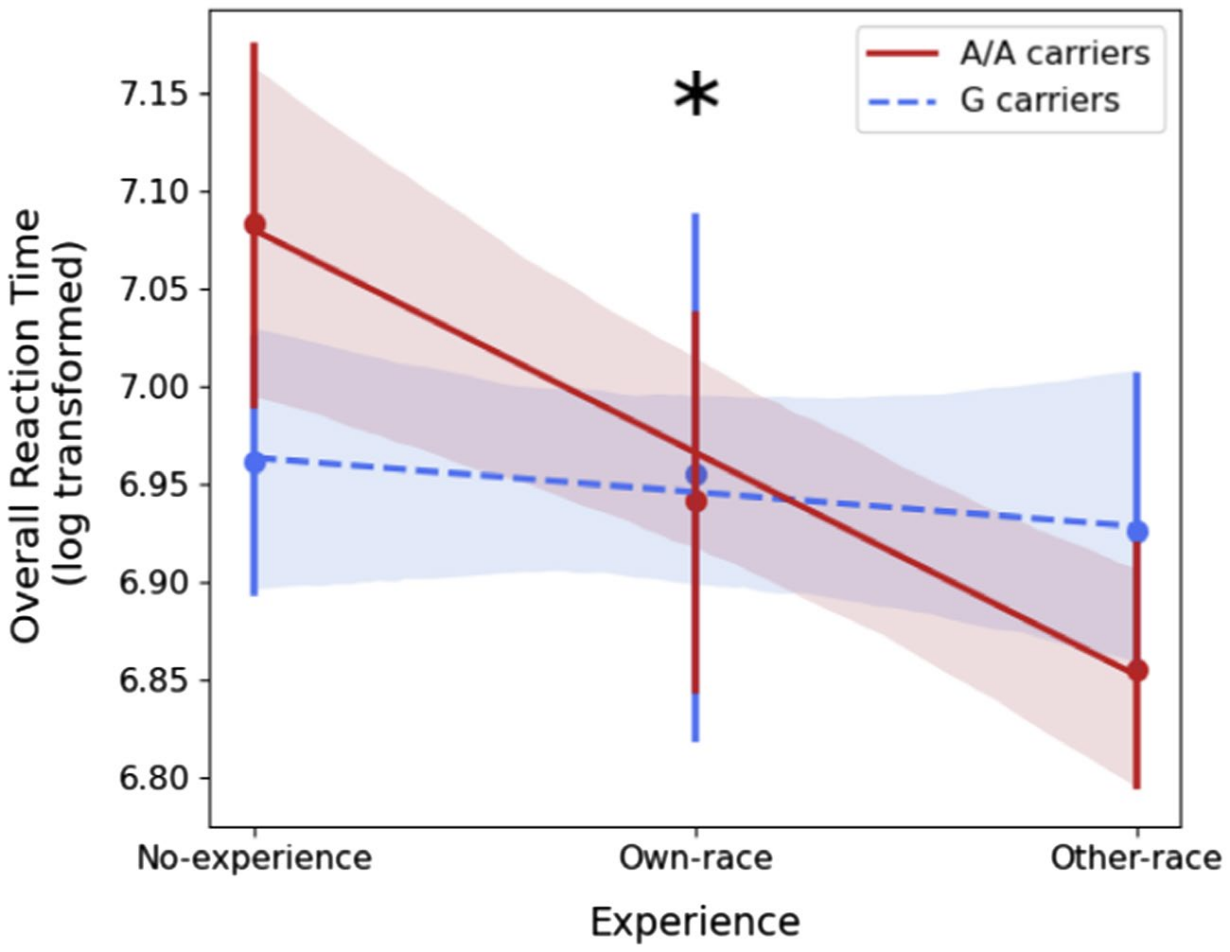
Regression lines representing the association between ORT and Experience for the two Genetic Groups, resulting from the multiple linear regression analysis. Circular markers indicate the mean, vertical bars indicate the 95% confidence intervals. Shaded areas represent the confidence intervals for the regression estimated by bootstrap. The asterisk indicates that the correlations between ORT and Experience for the two Genetic Groups are statistically significant (*p* < 0.05).

Descriptive statistics of the ORT for the two Genetic Groups and the three Experience groups are reported in Table 1. A multiple linear regression was calculated to predict ORT based on Genetic Group (A/A, G carriers) and Experience (No-experience = 0, Own-race = 1, Other-race = 2) and their interaction. A significant regression equation was found [*F*(3, 85) = 6.323 *p* < 0.001], with an *R*^2^ of 0.18 (see Table 2). A significant interaction of Genetic Group and Experience (*t* = 2.48, *p* = 0.015), as well as main effects of both Genetic Group (*t* = −2.17, *p* = 0.033) and Experience (*t* = −4.29, *p* < 0.001) emerged. Post-hoc analysis revealed no significant difference in the ORT between A/A carriers and G carriers [*t*(85.13) = 0.41, *p* = 0.685, Cohen’s *d* = 0.09], and a significant correlation between ORT and Experience [*ρ*(87) = −0.33, *p* = 0.002]. A significant correlation between Experience and ORT was found for the AA group [*ρ*(44) = −0.52, *p* < 0.001], but not for the G carriers [*ρ*(41) = −0.10, *p* = 0.533]. The difference in correlations was significant (*z* = −2.17, *p* = 0.015, see Figure 3).

**Table 1:** Distribution of the samples in the two genetic groups and types of early child caregiver experience.

	Child caregiver Experience	*N* (% of genetic group)	Overall reaction time [ms] *M* (SD)
AA (*N* = 46)	No-experience	19 (41.3)	1217.33 (254.76)
	Own-race	6 (13.0)	1043.21 (145.96)
	Other-race	21 (45.7)	958.72 (138.07)
G (*N* = 43)	No-experience	17 (39.5)	1065.05 (150.23)
	Own-race	8 (18.6)	1067.42 (217.94)
	Other-race	18 (41.9)	1032.52 (177.96)

**Table 2:** Results of the multiple linear regression to investigate the effects of Experience, Genetic Group, and their interaction on ORT.

Variable	*β*	95% Confidence interval [0.025, 0.975]	SE	*t*	Value of *p*
Intercept	7.08	[7.01, 7.15]	0.04	190.70	<0.001
Genetic Group [G carriers]	−0.12	[−0.22, −0.01]	0.05	−2.17	0.033
Experience	−0.11	[−0.17, −0.06]	0.03	−4.29	<0.001
Experience × Genetic Group [G carriers]	0.10	[0.02, 0.17]	0.04	2.48	0.015

## Discussion

The present study aimed to investigate the role of both genetic and environmental factors on other-race face recognition where we examined the rs53576 OXTR genotype and early caregiver experience in Singaporean adults. Here, we proposed the inclusion of a genetics perspective to be considered in investigating face categorization.

First, a significant interaction effect of Genetic Group with Experience was found in the present study, supporting Hypothesis 1. A possible explanation for this finding is the difference in prosociality between A/A homozygotes and G carriers. As discussed earlier, the G allele has previously been associated with adaptive prosocial traits. Hence, it is possible on average, G carriers have greater interracial contact with others and derive perceptual expertise from their social interactions as opposed to early caregiver experience compared to A/A homozygotes. As such, early caregiver experience constitutes a more significant source of interracial contact and exposure to other-race faces for A/A homozygotes and forms a significant avenue through which A/A homozygotes derive perceptual expertise in face categorization by race. These findings suggest that the environment—early caregiver experience—may be able to compensate for a profile of less adaptive social traits and characteristics that have previously been associated with A/A carriers of rs53576. Overall, these findings also strongly suggest the importance of applying a genetics perspective to examining face categorization by race given the significant gene–environment interaction discovered in the present study which align with previous studies indicating gene–environment interactions between OXTR genotype and environmental factors (see for example Esposito et al., 2017a,b).

Second, a significant correlation between Experience and categorization reaction time was found in the present study where individuals with other-race child caregiver experience had the fastest categorization reaction times in the A/A homozygotes. This finding is in line with previous findings in Rennels et al. (2017) and Tham et al. (2018) that the face processing system can be flexible to changes in caregiver experiences in an infant’s natural environment. Notably, Tham et al. (2018) found some evidence indicating that Chinese infants with additional other-race (Malay; a high frequency race in Malaysia) caregivers showed better recognition for other-race faces than infants without additional other-race caregivers in Malaysia. In terms of the frequency of Javanese as an other-race in Singapore, Singapore has a relatively high non-resident population (1.47 million as of June 2021; Singapore Department of Statistics, 2021) with 252,600 foreign domestic workers, making up 18.7% of the non-resident population (Ministry of Manpower, 2020) where it has been reported that one in five Singaporean households hires a foreign domestic worker (Awang and Wong, 2019) and about half of these foreign domestic workers are Indonesian (Devaraj, 2020). Hence, Javanese can be considered a relatively high frequency other-race in Singapore. In addition, Malays constitute the second largest racial group in Singapore where the Javanese are one of the Indonesian ethnic groups which migrated to Singapore and people with Javanese ancestry in Singapore have largely assimilated and are regarded as part of the Malay racial group (Tham, 1993). As such, in the context of the relatively large population of Javanese foreign domestic workers and Malay individuals in Singapore, an individual needs to be able to individuate and recognize their own other-race child caregiver apart from other Javanese faces, possibly leading to greater perceptual expertise derived from early experience with individuation and categorization of Javanese faces. This aligns with previous findings that early experience contributes to the ability to categorize faces by social categories, such as race. Hence, early experience with individuating an other-race face could result in more efficient cognitive processes involved in face categorization and consequently, faster reaction times for race-based categorization.

Majority of the studies on face processing have been conducted in predominantly monoracial populations living in racially homogeneous environments who generally lack experience with other-race faces (Valentine and Endo, 1992; Levin, 1996; Zhao and Bentin, 2008; Feng et al., 2011) and there are fewer studies looking at participants from multiracial societies, indicating that face processing in populations with greater experience with faces of different categories—specifically, race—is not well understood. Given the multiracial societal context that the study was conducted in—Singapore’s resident population consists of 74.2% Chinese; 13.7% Malay; 8.9% Indian; and 3.2% other races (Singapore Department of Statistics, 2021), the findings of the present study suggest that early childhood experience involving interracial contact appear to influence face categorization and future studies leveraging on the nature of multiracial societies can help to provide greater insight in investigating this phenomenon. Findings in the present study align with previous findings indicating better face recognition abilities in infants with other-race caregivers (Tham et al., 2018). Extending from this role of early childhood experience with other-race faces, another implication of these findings could be an important consideration during child development, especially in light of findings that racial categorization of faces has been associated with implicit racial bias in children (Setoh et al., 2019) and that training children can reduce implicit racial bias (Xiao et al., 2015).

### Limitations and Future Directions

However, there are a number of limitations to the study. Firstly, early caregiver experience with a child caregiver was simply measured in terms of absence of child caregiver or presence of other- or own-race child caregiver. A number of other factors, such as time spent with the child caregiver and quality of the relationship, could possibly influence the experience that individuals would have. Hence, future studies can investigate these factors to better understand how these factors may moderate the effect of early caregiving experience on race-based face categorization. The present study did not assess prosociality directly and further studies specifically comparing prosociality traits between rs53576 genotypes would allow for greater clarity of the underlying mechanisms of the gene–environment interaction on face categorization found in the present study. For example, future studies can make use of measures of prosociality, such as behavioral coding (e.g., Kogan et al., 2011) or self-reports, such as the Tridimensional Personality Questionnaire (e.g., Tost et al., 2010), and look into possible associations with the interaction experiences of adults with members of other races in their daily lives. By investigating the relationships between these factors between genetic groups, these studies can further strengthen the possible explanation that greater prosociality in G allele carriers facilitates greater experience interacting with members of different social categories in daily life compared to A/A homozygotes, resulting in early caregiving experience with other-race child caregivers constituting a less significant source of interracial contact for the G allele carriers relative to A/A homozygotes.

Secondly, early experiences with a child caregiver were measured retrospectively by asking adult participants to recall their childhood experiences. While it may be difficult to meaningfully quantify the extent of one’s own vs. other-race exposure in a multicultural society like Singapore, in terms of the other-race caregiver experience in the present study, the other-race child caregivers in the study are foreign domestic workers who live in with the household that they work for, which implies a significant majority of time, possibly close to 24-h exposure a day, since they are living in the same household. Future studies can seek to further strengthen current findings by employing a longitudinal design examining race-based face categorization in children as they develop, where the possible explanations that we proposed for our findings can be tested in terms of the relationship between the ability to individuate one’s other-race child caregiver and the categorization response. Both the experiences an individual has with own and other-race individuals as well as the specific timeframe in life during which the early caregiving experience and other experiences with own and other-race individuals occurred may also contribute to differences in face categorization abilities and future studies can also look into whether the age at which the child caregiver experience occurred influences face categorization. These studies will also contribute to a more accurate depiction of the duration and timing of exposure to other-race child caregivers that must elapse for significant differences in categorization responses with individuals without caregiving experience to arise.

Thirdly, the sample in the present study did not have an equal distribution of participants for each OXTR genotype group as specific early life experiences and OXTR genotypes are not easily determined during participant recruitment. The sample was also made up of more females than males. Future studies can look to expand on and validate present findings by recruiting a larger sample.

## Conclusion

The present study found that early child caregiver experience affects the speed of categorization response times, suggesting that perceptual expertise could play a role in face categorization abilities in adults of a multiracial society. There was also preliminary evidence pointing toward gene–environment interactions influencing speed of face categorization, specifically the rs53576 OXTr genotype. Such gene–environment interactions suggest that environmental factors could help to compensate for genetic predispositions relating to face categorization. Findings from the present study suggest that studies on face categorization need to take into account the demographic of the population individuals are exposed to as well as the contributing role of genetics, especially given the vast differences in both these factors in societies around the world.

## Data Availability Statement

The datasets presented in this study can be found in online repositories. The names of the repository/repositories and accession number(s) can be found at: (DR-NTU) https://doi.org/10.21979/N9/IWGQ1M.

## Ethics Statement

The studies involving human participants were reviewed and approved by Nanyang Technological University Institutional Review Board. The patients/participants provided their written informed consent to participate in this study.

## Author Contributions

PS and GE conceptualized the study. PS collected the data. MT and JF analyzed the genetic data. AB, MB, and GE analyzed the data. MN, AB, MB, and GE wrote the first draft. All the authors reviewed and edited the submitted version of the article.

## Funding

This research was supported by NAP SUG 2015 (GE), Singapore Ministry of Education ACR Tier 1 (RG55/18, GE and AL) and Singapore Ministry of Education Social Science Research Thematic Grant (MOE2016-SSRTG-017, PS).

## Conflict of Interest

The authors declare that the research was conducted in the absence of any commercial or financial relationships that could be construed as a potential conflict of interest.

The handling editor declared a past co-authorship with one of the authors GE.

## Publisher’s Note

All claims expressed in this article are solely those of the authors and do not necessarily represent those of their affiliated organizations, or those of the publisher, the editors and the reviewers. Any product that may be evaluated in this article, or claim that may be made by its manufacturer, is not guaranteed or endorsed by the publisher.
